# Rural Environments and Community Health (REACH): a randomised controlled trial protocol for an online walking intervention in rural adults

**DOI:** 10.1186/1471-2458-14-969

**Published:** 2014-09-18

**Authors:** Braden L Mitchell, Nicole R Lewis, Ashleigh E Smith, Alex V Rowlands, Gaynor Parfitt, James Dollman

**Affiliations:** Exercise for Health and Human Performance Group, Sansom Institute for Health Research, University of South Australia, Adelaide, Australia; University of South Australia, CEA-14, GPO Box2471, Adelaide, South Australia 5001 Australia

**Keywords:** Physical activity, Walking, Pedometer, Cardiovascular risk, Online intervention, Lifestyle intervention, Health behaviour

## Abstract

**Background:**

Rural Australian adults are continually shown to be insufficiently active with higher prevalence of lifestyle-related diseases associated with physical inactivity compared to urban adults. This may, partly, be attributable to the challenges associated with implementing community-based physical activity programs in rural communities. There is a need for broadly accessible physical activity programs specifically tailored to the unique attributes of rural communities. The aim of the Rural Environments And Community Health (REACH) study is to evaluate the effectiveness of an online-delivered physical activity intervention for increasing regular walking among adults living in rural areas of South Australia.

**Methods/Design:**

This is a randomised controlled trial. The intervention is 12-weeks with a 12-month follow-up. Participants will be insufficiently active, aged 18 to 70 years and randomly assigned to either Control or Intervention group. Participants receive a pedometer, but only the Intervention group will receive access to the purpose built REACH website where they will report steps taken, affect and ratings of perceived exertion during daily walking. These variables will be used to establish individualised step goals for increasing walking. Control participants will receive a paper diary to record their variables and generic incremental step goals.

The primary outcome measures are time spent in sedentary, light and moderate-to-vigorous intensity physical activity, measured by accelerometry. Secondary outcomes include 1) health measures (anthropometric and physiological), 2) psychological well-being, 3) diet quality, and 4) correlates of physical activity (exercise self-efficacy and physical activity environments). Measures will be collected at baseline, post-intervention, 6-month and 12-month follow-up.

**Discussion:**

This protocol describes the implementation of a trial testing the effectiveness of an online resource designed to assist rural Australians to become more physically active. The outcomes of this study will guide the efforts of health promotion professionals by providing evidence for a relatively inexpensive, widely accessible and effective method for increasing physical activity that can be utilized by anyone with access to the internet. Findings may indicate future directions for the implementation of physical activity and other health related interventions in rural communities.

**Trial registration:**

Australian New Zealand Clinical Trials Registry: ACTR12614000927628 (registered 28 August 2014).

## Background

More than half of Australia’s adult population does not participate in sufficient amounts of physical activity to infer positive benefits to health [1]. Of particular concern are rural Australian adults, who are not only more likely to report being physically inactive [1], but also suffer disproportionately from lifestyle-related diseases such as cardiovascular disease and type-2 diabetes mellitus [2, 3].

Increasing physical activity provides an effective means of reducing one’s risk of lifestyle-related disease [4–6]. Furthermore, evidence has consistently indicated that regular participation in physical activity benefits many other physiological outcomes (e.g. cardiovascular fitness, immune function, muscle strength and body composition), as well as improving psychological wellbeing (mood, self-esteem, depression and anxiety) [7–9]. It is widely accepted that, to infer positive effects on health, adults should accumulate at least 30 minutes of moderate or vigorous physical activity on at least five days of the week [10, 11]. More recently, recommendations of achieving at least 10,000 steps per day have gained significant momentum as an easily measureable indicator of sufficient physical activity [5, 12].

Despite the well-documented benefits of physical activity for health, rural Australian adults remain less physically active than their urban counterparts [1]. As much as 63% and 65% of rural men and women, respectively, do not report sufficient levels of physical activity, compared to just 55% and 60% respectively in major cities [13]. In fact, according to Vaughan and colleagues [14], as little as 25% of rural adults attain the recommended amounts of physical activity. This may be, in part, attributable to the challenges of implementing community-based physical activity programs in rural communities where accessibility to services and facilities can be limited [15, 16]. As such, previous suggestions for improving physical activity levels in urban environments, for example cycling to work or walking to the local shops, may not be feasible in a rural setting. Similarly, rural infrastructure may not be conducive to physically active lifestyles, where common urban activities such as cycling to work may require individuals to share major arterials with high speed traffic and heavy vehicles. Thus, there is a need for safe and broadly accessible physical activity promotion interventions which are specifically tailored to the unique lifestyles and environments of rural Australian adults.

The purpose of this paper is to describe the protocol of the REACH (Rural Environments and Community Health) 12-week physical activity program, which has been specifically designed to test the effectiveness of an online-delivered walking intervention for increasing regular walking among adults living in rural areas of South Australia. The trial also aims to assess the effects of the walking intervention on markers of cardiovascular risk and to identify psychological, social and environmental predictors of a sustained response to the intervention. We hypothesise that the online delivered walking intervention will improve physical activity levels and improve cardiovascular risk profiles. We further hypothesise that psychological, social and environmental factors will predict longer term response to the online delivered walking intervention, both independently and interactively.

### Theoretical framework

The REACH intervention is based on Bandura’s [17] *social cognitive theory*. When applied to the context of physical activity interventions, social cognitive theory posits that interventions which include intrapersonal mediators (e.g. goal-setting, self-monitoring and self-efficacy), social mediators (e.g. family and peer support), and environmental mediators (e.g. access to facilities and opportunities) are most effective at eliciting a positive and sustained response [18–20]. Furthermore, concepts from Locke and Latham’s [21] *goal setting theory* will be integrated into the intervention. This will be achieved by 1) developing *goal acceptance* (participants will be provided information regarding the importance of physically active lifestyles for health and wellbeing, thus, placing emphasis on achieving step goals); 2) incorporating *goal specificity* (step goals for the intervention group will be individualised, taking into account how they are feeling, their ability to achieve previous goals and physical capabilities); 3) providing *difficult goals* (step goals will be set high enough to provide a challenge and not be seen as too easy, but low enough to be attainable as to not discourage motivation); and 4) providing *feedback* (the REACH website graphs weekly step count averages to show changes over the 12-week program).

## Methods/Design

### Participants and setting

The REACH study is a randomised controlled intervention trial. Approximately 300 participants will be recruited from rural South Australia and randomised into either the Intervention or Control group. Table 1 outlines the inclusion and exclusion criteria for the study. For the purposes of this study, ‘rural’ is defined as a Remoteness Area, identified by the Australian Bureau of Statistics (ABS), as predominantly outer regional, remote or very remote. The Riverland and Yorke Peninsula regions of South Australia have therefore been selected as recruitment regions. According to the ABS, 86.3% and 83.1% of the Riverland [22] and Yorke Peninsula [23] are classed as outer regional, respectively, satisfying the aforementioned criteria. The Riverland has an approximate population of 42,000 people [22] and an average Socio-Economic Indexes for Areas (SEIFA) score of 914.14 [24], indicating an area of relative socio-economic disadvantage. The Yorke Peninsula has an approximate population of 27,500 [23] people and an average SEIFA score of 924.44 [24], indicating similar socio-economic disadvantage. The dominant employment sectors of these two regions are indicative of those associated with rural Australia, with the main employment sector in the Riverland being fruit and nut tree growing [25], while dry land farming (crops and livestock) serves as the dominant employment sector in the Yorke Peninsula [26].

**Table 1:** **Inclusion and exclusion criteria**

Inclusion criteria	Exclusion criteria
• Aged 18-70 years	• Non-resident or resident <12-months of target regions prior to study start
• Self-reported resident of target regions for minimum of 12-months prior to study start	• Relocation of primary residence outside of the target regions during the study period (participants will be unenrolled from the study from that point on)
• Insufficiently active (engaging in fewer than 20 bouts of physical activity of at least 30 minutes) over the previous month	• Pregnant or intending to become pregnant during 12-month study period
• Access to the internet	• Physical or psychological condition (i.e. cognitive impairment) that may impede full participation in the study
• Deemed medically safe to participate in unsupervised moderate-intensity physical activity by satisfaction of stage one of the Adult Pre-Exercise Screening System (or review by an Accredited Exercise Physiologist)	
• Sufficient English language skills and cognitive ability to allow full participation in the study	
• Able to provide written informed consent	

### Funding and ethical approval

Funding for this project has been provided by a grant from the South Australian Cardiovascular Research Development fellowship scheme of the National Heart Foundation of Australia (Project ID: 0000035126). All participants in the study will be provided with information pertaining to the measures and processes of the study, following which informed written consent will be attained. This study protocol has received ethical approval from the University of South Australia Human Research Ethics Committee (no. 0000031466) and is registered with the Australian and New Zealand Clinical Trials Registry (Trial no. ACTRN12614000927628).

### Study design

#### Recruitment and screening

**Figure 1:**
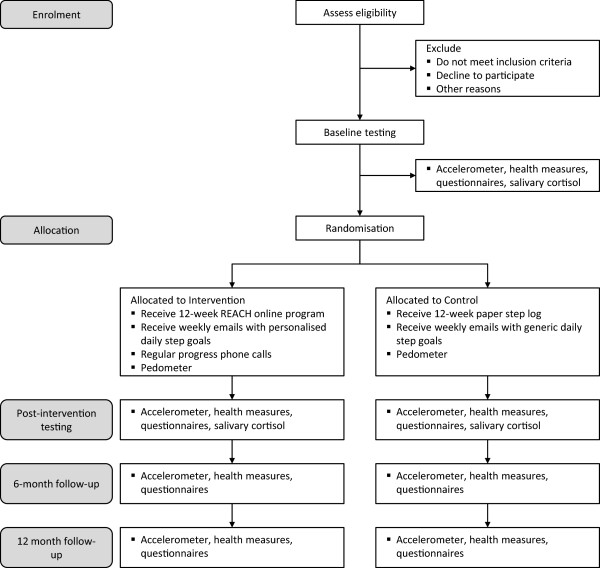
**Outline of REACH study protocol.** Health measures include anthropometry, resting blood pressure, blood chemistry and cardiovascular fitness. Questionnaires include psychological well-being, diet quality, sleep quality, correlates of physical activity and demographics.

Outline for the overall study protocol is presented in Figure 1. Participants will be recruited via local newspaper advertisements, rural organisations (e.g. Country Women Association and SA Farmers Federation), local radio, school newsletters, local businesses and sporting/community clubs. Potential participants will initially be screened via telephone to determine eligibility. Eligible and willing participants will be provided with further information regarding the study and booked into an initial testing session. Written consent will be attained and pre-exercise screening completed during the initial testing session. Pre-exercise screening will be conducted using stage one of the Australian Adult Pre-exercise Screening System (APSS) [27]. Stage one of the APSS is designed to identify individuals with signs or symptoms of underlying disease, or those who may be at high risk of adverse events during exercise. Any participants who answer “Yes” to any questions in stage one will be assessed by an Accredited Exercise Physiologist for suitability to participate in unsupervised moderate-intensity exercise.

#### Randomisation and blinding

Participants will be randomised into either the Control or Intervention arm using a cluster randomisation technique. Participants with complex medical conditions will be reviewed by an Accredited Exercise Physiologist (BM, NL) and, if deemed to require increased monitoring, will be placed in the Intervention group for safety. It is not possible to blind key researchers (BM, NL) to participants’ group allocation as they will be performing information sessions prior to the intervention. Similarly, it is not possible to blind participants to group allocation due to the nature of the study design. Six other research assistants performing testing will be blinded to the participants’ group allocation.

### Protocol

#### Testing procedure

All participants will attend four baseline sessions, each one week apart. Session one will last approximately 20 minutes, while the remaining sessions will last approximately 60 minutes. At the first session, held during the evening, participants will be provided with an accelerometer and instructed in the correct measurement protocol. During session two, to be held early in the morning, health measures (anthropometry, blood pressure and blood chemistry) will be taken and participants will complete a dietary questionnaire. At the end of this session, participants will be provided with, and instructed in, the use of a Salivette saliva sampling kit. Session three, held during the afternoon, will comprise the completion of a further three questionnaires assessing psychological wellbeing, sweet-drink intake and general health/demographics. All questionnaires will be self-administered using a computer-based survey system. Two three-minute walking tests will also be completed. Session four, performed at baseline only, provides an information session for the participants about the details of the intervention, designed separately for Intervention and Control groups. The Control group will be instructed on the importance of participating in regular physical activity as per the Australian Physical Activity Guidelines [10], how to setup and use their pedometer (Yamax DW700; Yamasa Tokei Keiki Co. Ltd., Tokyo, Japan) and tips for incorporating physical activity into daily life. The Intervention group, in addition to the information provided to the Control group, will be shown how to use the REACH website, including how to log their steps and other information. Measures will be repeated following the 12-weeks of the intervention period, and at 6-months and 12-months follow-up (i.e. 3-months and 9-months from end of the intervention). Participants will be recruited from three intervention regions: the Riverland; the Copper Coast (Upper Yorke Peninsula); and the Lower Yorke Peninsula. In each location, testing sessions will be provided in four different towns on different days. This is to minimise participant burden by providing a number of opportunities to participate around their usual schedule and reduce distances needed to travel. Testing will begin in the Riverland, followed by the Copper Coast and then the Lower Yorke Peninsula. Testing in the second and third regions will begin during the information session (session four) of the preceding region.

#### Intervention protocol

The REACH intervention involves three key components, the REACH website, weekly step goals and ongoing progress support provided in the form of telephone interviews. The specifics of, and access to, these three key attributes are dependent on group allocation. Participants in both groups will use a pedometer to monitor the number of steps taken each day. Those in the Control group will be provided with a standard logbook to record their steps. The Intervention group will be provided access to the REACH website. The website allows the participant to record their steps, which are then depicted on a graph, to provide feedback to the participant in an easy to read visual format. In addition to steps taken, participants will also record their affective state (how they are feeling) and ratings of perceived exertion (RPE). Intervention participants will input these on the REACH website, while control participants will make note in their logbooks. The REACH website also provides an online forum where participants can share their experiences and tips with others in the program and provide peer support. A virtual notice board will collate information on community events and services, walking groups and other relevant region-specific information. This aspect of the website is designed to provide ready access to supervised and socially focussed opportunities to be physically active in their local communities. The website will also provide ready access to healthy eating guidelines based on the recommendations of The Australian Guide to Healthy Eating [28].

Ongoing progress support phone calls will be provided to the Intervention group only. These phone calls will occur in weeks two, three, four, six, eight and twelve. The purpose of gradually reducing contact with the participant is to foster a milieu of independence and increased self-efficacy within the participant. These phone calls will be approximately 15 minutes in length and will cover aspects related to progress, website use, perceived barriers, injury/illness and tips for increasing physical activity through incorporation into daily routines. The Control group will receive one phone call during week two to ensure they understand the requirements of the study, but will not receive any additional progress phone calls thereafter.

#### Step goals

For the Intervention group, personalised step goals will be created using individuals’ RPE, affect and ability to achieve their previous week’s goals. RPE is determined using Borg’s [29] 6–20 Ratings of Perceived Exertion (RPE) scale which serves to numerically quantify the perceived effort, strain, discomfort and/or fatigue experienced during physical activity by the individual undertaking it. RPE is a widely used construct, having been previously used to prescribe target exercise intensities in both healthy adults [30, 31] and clinical populations [32–35]. Previous research has suggested that RPE may be beneficial in populations where maximal exertion is contraindicated [36]. Affective state (i.e. feeling good/bad) has been shown to significantly influence exercise behaviour and motivation [37]. Participants will rate their daily affective state (i.e. how they feel [good or bad]) when they get out of bed on an 11-point Likert scale ranging from +5 (‘very good’) to -5 (‘very bad’) [38].

Researchers on the REACH team will use these inputs (daily steps, RPE and daily affect) to generate individually tailored step goals for the following week. Participants are encouraged to maintain an exertion between RPE-11 (light) and RPE-13 (somewhat hard) on the RPE scale, the ‘bandwidth’ within which people have reported the most positive response to exercise [39–41]. Step goals will be provided in the form of a ‘target range’. The first week will serve as a baseline from which to set the first goals, with a 5-10% increase per week in step goals. If participants do not meet their previous week’s step goals on most days, the goals for the preceding week will not change.

Step goals for the Control group will be generic across all control group participants. Step goals will start at 5,000 to 6,000 steps in week one and progress in weekly increments of 500 steps until reaching the recommended 10,500 to 11,500 steps [5, 12] in week 12 of the intervention.

### Outcome measures

The primary outcomes are daily minutes spent participating in sedentary, light intensity and moderate-to-vigorous intensity physical activity. The mean daily activity intensity will also be assessed. To objectively measure these outcomes, participants will wear a tri-axial wrist-worn accelerometer (GENEActiv; Activinsights Ltd, Cambridgeshire, United Kingdom), on their non-dominant wrist, for a period of seven consecutive days (five week days and two weekend days). Matthews and colleagues [42] argue that seven days of monitoring provides sufficient data to achieve intra-class correlations of greater than 80%, and ensures the inclusion of both week day and weekend day behaviours. The GENEActiv’s design is unobtrusive and water proof, minimising the need for removal and therefore maximizing compliance with continued wear over the sampling period. Output data will be analysed using cut-points previously established by Esliger and colleagues [43] from 60-second epochs using a custom-made algorithm in Microsoft Excel. Non-wear time will be assessed automatically using a custom-made algorithm. Monitors are to be worn for no fewer than sixteen hours in a day for the day to be classed as valid. A minimum of three valid week days and one valid weekend days will be required (four days in total) for the overall monitoring period to be acceptable. The GENEActiv shows high technical reliability, with intra- and inter-device variability of 1.8% and 2.4%, respectively [43], while its ability to discriminate between different intensities of physical activity is similar to other devices extensively used in physical activity research (GENEActiv: 0.90-0.93 [area under the curve] vs Actigraph GT1M: 0.94 vs RT3: 0.95) [43].

Secondary outcomes include:

#### Anthropometry

The following anthropometric measures will be taken: standing stretch stature; body weight and composition (Tanita BC-418; Tanita Corp., Tokyo, Japan); and waist and hip circumferences (Lufkin Executive Thinline 2 m W606pm; Apex Tool Group, LLC, Sparks, MD). All anthropometric measures will be taken in accordance with the International Standards for Anthropometric Assessment [44] by the same research personnel at all time periods. Body composition, expressed as percentage body-fat, will be assessed using single frequency (50 Hz) bioelectrical impedance analysis scales (Tanita BC-418). Assessment of body composition by the Tanita BC-418 shows strong correlation with measures by dual-energy x-ray absorptiometry (Pearson’s r = 0.87) with excellent test-retest reliability (intra- and inter-day coefficients of variability of 0.8-1.4% and 2.3-3.7%, respectively) [45]. A minimum of two measures will be taken for each outcome. A third measure will be required if initial measures differ by ≥1%. Final measures will be reported as the mean of two measures, or if a third was required, the median of all three.

#### Blood pressure

Resting blood pressure will be measured in a seated position using an automated sphygmomanometer (GE Carescape V100; GE Healthcare, Little Chalfont, United Kingdom) after participants have been rested for a period of five minutes. A minimum of two measures will be taken, with at least two minutes rest, with the cuff removed, between measures. Additional measures will be required if either systolic or diastolic pressures differ by >5 mmHg, to a maximum of four measures. Final resting blood pressure will be reported as the mean of the two closest measures.

#### Blood chemistry

Blood cholesterol, triglycerides and fasting plasma glucose will be assessed from an overnight fasted (minimum eight-hours) fingertip blood sample. Samples will be collected by research personnel experienced in fingertip blood sampling techniques and analysed on-site using Cholestech LDX (Alere Inc., Waltham, MA) technology. The Cholestech LDX analyser shows excellent agreement with laboratory analyses [46] and has been validated as an alternative to laboratory analyses for assessing the presence of metabolic syndrome [47].

#### Psychological measures

Psychological well-being will be assessed using the short-form Depression, Anxiety and Stress Scale (DASS-21). The DASS-21 is a self-administered questionnaire and comprises 21 items that are separated into three sub-scales representing three domains of psychological well-being: depression; anxiety; and psychological stress. Each sub-scale can be represented by a numerical score or categorised into five categories: normal; mild; moderate; severe; and extremely severe, as per the recommendations of Lovibond & Lovibond [48]. The DASS-21 has been validated for use in non-clinical populations as a tool for assessing symptomatology and has extensive empirical backing for validity and reliability, boasting Cronbach alpha coefficients of 0.88, 0.82 and 0.93 for the depression, anxiety and stress sub-scales, respectively [49].

Salivary cortisol will be assessed as a biomarker of psychological distress. There is emerging evidence that perturbations to cortisol’s underlying circadian rhythm may occur as a result of psychological or emotional distress. Seven saliva samples will be collected from each participant on a single week-day using Salivette collection devices (Sarstedt, Nümbrecht, Germany). Sample 1 (S1) is to be collected immediately on awakening, at 30 minutes and 60 minutes post-awakening (S2 and S3, respectively). In light of anecdotal evidence suggesting considerable difference in daily routines in rural populations, daily milestones rather than strict time points will be used to collect the remaining samples. These will be mid-morning (halfway between S3 and lunch), 30 minutes before lunch, 30 minutes before dinner and immediately prior to going to bed. Participants will be asked to abstain from smoking, consuming food or fluids (except water), brushing their teeth and strenuous physical activity for at least one hour prior to providing the sample. Participants will record the exact time of each sample on a log sheet. There is considerable evidence to suggest the use of hormonal contraceptives, hormone replacement therapies and cortisone based medications may alter cortisol regulation [50]. As such information regarding these factors will be collected by a research assistant from participants returning their samples. Analyses of samples will be performed using a standard enzyme-linked immunosorbent assay protocol (ELISA; Salimetrics LLC., State College, PA) at the University of South Australia. Parameters of the cortisol rhythm will be quantified based on recommendations from the literature [51, 52].

#### Diet quality

The Dietary Questionnaire for Epidemiological Studies (version 2) (DQES), developed by the Cancer Council of Victoria [53], will be used to assess dietary habits. The DQES is a 96-item questionnaire designed to assess food intake over the previous 12-months, and takes approximately 30-45 minutes to complete. It contains 74 items for which participants indicate how frequently they would have consumed the item on a 10-point scale ranging from “0 - Never” to “10 - 3 or more times per day”. Included in the questionnaire are three photographs of scaled portions of four foods and questions regarding the frequency of fruit and vegetable intake and other foods which do not readily fit the aforementioned intake frequency format (e.g. added sugar, breads and cheese). The 74 food items are separated into four groups: cereal foods, sweets and snacks; dairy products, meat and fish; fruit; and vegetables. Consumption of alcoholic beverages is assessed by a separate set of questions. The DQES has been validated for use in mid-age Australian women and young Australian adults [54].

To account for sweetened drink intake (soft-drink, sports drinks, energy drinks, fruit drinks and cordial) which is not assessed by the DQES, participants will be asked to fill out a 6-item questionnaire. For each sweetened drink category (full sugar soft-drink; sports/energy drinks; and fruit drinks/cordial) participants are to indicate, on average, how often they had consumed the drink in the previous 12-months on a 10-point scale using the same frequency increments as in the DQES. Participants will also indicate, when they consumed the drink, how much they usually consume on a 3-point scale consisting of “One glass (250 mL)”, “One can (375 mL)” or “One bottle (600 mL)”. Output data from the DQES will be amended post-hoc to account for nutrient contribution attributable to the intake of sweet drinks.

#### Fitness

Cardiovascular fitness is to be assessed by a three minute walk test, performed at a self-regulated intensity of RPE-13. Walking distances will then be used to predict maximal oxygen consumption (VO_2max_) using the equation of Cao et al. [55] which is strongly correlated with objectively measured maximal oxygen consumption (R = 0.84, SEE = 4.57mL▪kg^-1^▪min^-1^) during maximal incremental cycle tests.

#### Sleep quality

Aspects of sleep quantity and quality will be assessed using questions four and six from the Pittsburgh Sleep Quality Index [56], while questions from Maislin and colleagues [57] will be used as an indicator of sleep disordered breathing symptomatology.

#### Correlates of physical activity

Exercise self-efficacy and the salient aspects of the physical environment will also be assessed from questions adapted from Nigg and Riebe [58] (barrier self-efficacy), Schwarzer and colleagues [59] (relapse self-efficacy) and De Bourdeaudhuij, Sallis and Saelens [60] (physical activity environment).

#### Demographics

Basic demographics including age, gender, ethnicity, highest attained education, occupation and marital status, as well as perceived general health, family history of disease and smoking status will be collected using a customised questionnaire.

### Statistical considerations

#### Sample size

A priori power calculations indicated 106 participants are required per group (n = 212) to detect a medium effect size (Cohen’s d = 0.5), at 80% power, with significance level set at α = 0.05 and the assumption that the predictors will contribute an R^2^ of 0.5. Given a 30% attrition rate from a recent pilot study (unpublished) a total sample size of 300 will be targeted.

#### Statistical plan

Data will be collated and manipulated using Microsoft Excel 2010 (Microsoft Corporation, Redmond, WA). Statistical analyses will be performed using STATA Data Analysis and Statistical Software version 12 (StataCorp LP, College Station, TX) and SPSS version 21 (IBM corp., Armonk, NY). Significance levels will be set at α = 0.05. Distributions of all variables will be assessed using the Shapiro-Wilk test, with appropriate normalization procedures applied where required. Independent samples t-tests will be performed on baseline measures to test for differences in demographic outcomes. Change scores for all outcomes will be calculated and compared between randomisation groups using parametric or non-parametric analyses as appropriate for the data distributions. Random effects mixed modelling will be used with time (0, 12, 26 and 52 weeks) and group allocation (Intervention and Control) as the fixed factors. A time by group interaction term will be used to formally test the aims of the study.

## Discussion

Engagement in regular physical activity is consistently shown to positively impact both physical and psychological well-being [4–9]. Despite this, rural Australian adults still report significantly higher levels of physical inactivity [1] and also suffer disproportionately from lifestyle-related diseases shown to be associated with insufficient physical activity [2, 3]. The achievement of 10,000 steps per day is widely seen as a marker of sufficient physical activity to benefit one’s physical health. According to Choi and colleagues [12], for most people, the 10,000 step goal is not achievable through daily routines, often finishing with a 4,000 to 6,000 step deficit. Estimates suggest that walking can provide an individual the opportunity to achieve 100-150 steps per minute [12]. Simply walking for 30-60 minutes a day can therefore help individuals to achieve their recommended 10,000 steps in an affordable and accessible way, with the concomitant increase in moderate-intensity physical activity to positively impact health.

The REACH study will contribute significantly to the literature as it will use RPE and daily affective states to develop individualised, incremental step goals relative to each individual’s baseline values. Thus, the intervention will be tailored to the individual’s physical abilities and capacity for physical activity. The study outcomes will guide the efforts of health promotion professionals by providing evidence for a relatively inexpensive, widely accessible and effective form of exercise. The study’s unique online delivery allows the program to be accessible to anyone with an internet connection. This is an important distinction for rural communities where access to services and facilities are restricted compared to urban populations.

## Authors’ information

BM is a PhD candidate within the School of Health Sciences at the University of South Australia and is an Accredited Exercise Physiologist with Exercise and Sports Science Australia (ESSA). NL is project manager and is an Accredited Exercise Physiologist with ESSA. AS is a Postdoctoral Research Fellow, AR a Senior Research Fellow and both GP and JD are Associate Professors. All authors are based in the Exercise for Health and Human Performance Group, within the School of Health Sciences, at the University of South Australia.

## Abbreviations

REACH: Rural Environments and Community Health
ABS: Australian Bureau of Statistics
SEIFA: Socio-Economic Index for Areas
APSS: Adult Pre-exercise Screening System
RPE: Rating of perceived exertion
DASS-21: Short-form depression, anxiety and stress scale
DQES: Dietary Questionnaire for Epidemiological Studies.
